# Removing a Bent Femoral Intramedullary Nail Cost Effectively: A Case Report

**DOI:** 10.5704/MOJ.2107.024

**Published:** 2021-07

**Authors:** SH Sa’aid, MY Bajuri, FN Dzeidee-Schaff, MH Abdul-Suki

**Affiliations:** Department of Orthopaedic and Traumatology, Universiti Kebangsaan Malaysia, Kuala Lumpur, Malaysia

**Keywords:** bent intramedullary nail, nail extraction, femoral shaft fracture

## Abstract

A bent intramedullary (IM) nail becomes challenging and technically demanding to the orthopaedic surgeon for nail extraction. A broken nail can be easily removed through the fracture site. However, a bent nail has to be broken before it can be removed. Several studies and case reports outline the strategies and techniques for removing a bent IM nail. However, there is a paucity of guidelines and standard protocol describing the best and inexpensive strategy. We report a case where two years following surgery for intramedullary nailing of the right femur, the IM mail was bent following secondary trauma. We used a technique based on the principles of an ability to fully cut the nail and extract it in two pieces by using a Jumbo cutter which is available in the orthopaedic armamentarium. This technique is simple yet economical, with the likelihood of causing less soft tissue damage and thermal necrosis.

## Introduction

Femoral shaft fracture is the most common injury following road traffic accidents and accounts for up to 37 per 100 000 patient-year, with a peak incidence in young adults. There is an increased risk of acquiring pulmonary complications such as fat embolism syndrome (FES), acute respiratory distress syndrome (ARDS) and pneumonia in delayed stabilisation of femoral shaft fractures, particularly in patients with multiple injuries^1^. The gold standard procedure preferred for the treatment of femoral shaft fractures is the intramedullary (IM) nail. The device is load sharing so that the stress shielding is less in IM nail and peri-implant fractures are less. The fracture hematoma and the cambium layer of periosteum which is needed for bony healing is preserved thus the union rate is high^2^. Although many studies report low levels of complications, one rare complication of IM nail is a bent nail following a secondary trauma to the previous fracture site^3^. A bent IM nail becomes challenging and technically demanding to the orthopaedic surgeon for nail extraction^3^. Several studies and case reports outline the strategies and techniques for removing a bent IM nail. However, there are no guidelines and standard protocols which describes the best strategy and it is often expensive and frequently unobtainable in most centres.

We report a case of a bent femoral nail following secondary trauma and the technique to remove it.

## Case Report

**Fig. 1:**
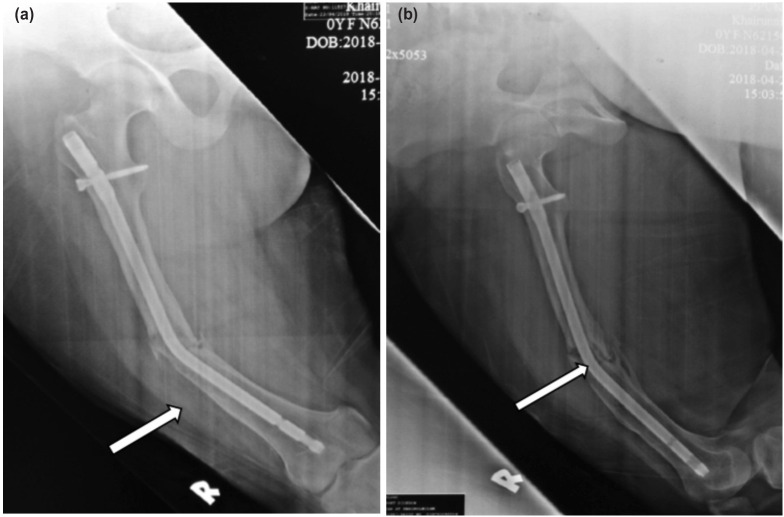
(a) Plain radiograph shown 30° varus angulation of femoral nail in AP view (b) and 30° anterior angulations in lateral view with the continuity of the nail was intact. It also shown that the femoral shaft fracture was not united with unicortical callus formation.

A 17-year-old Malay girl was involved in a road traffic accident in which she sustained a closed right femur fracture. She was treated with intramedullary nailing of the right femur. She was well until two years after the initial trauma when she met with another road traffic accident. The patient fell off her motorcycle and was brought to our centre with severe right thigh pain. Clinically, the right thigh was deformed with no obvious open wound. There was no other associated injury and the neurovascular status was intact. A plain radiograph shown 30° varus angulation of the femoral nail in the AP view and 30° anterior angulation in the lateral view with the continuity of the nail intact (Fig. 1). The fracture site was noted to be not united with minimal callus formation. It was decided to remove the nail surgically. The surgery was done under regional (spinal) anaesthesia with the patient being placed in the left lateral decubitus position. A skin incision was made by a lateral approach at the fracture site under image intensifier guidance. The tissue was divided layer by layer until the fracture site and the fibrous tissue around it was cleared up.

**Fig. 2:**
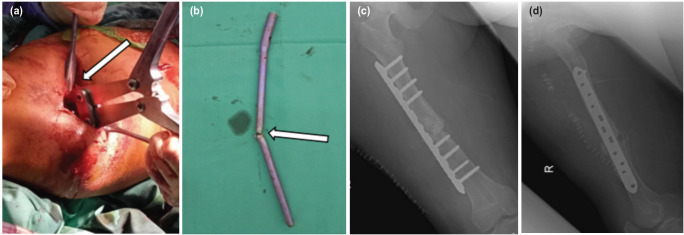
(a) Jumbo cutter was introduced at visible nail (b) and sectioned the nail in two parts with multiple bites. Appearance of complete extracted nail - two parts. (c, d) Immediate postoperative radiograph showing locking compression plate and bone graft was done after removal of bent nail.

The initial technique attempted was based on the principle of partially weakening the nail followed by manual straightening. The bent nail was partially resected using a jumbo cutter through the wound, then reduction was attempted using the bone holder forceps placed at the proximal and distal part of the fracture site and the strength was applied to straighten the bent nail. However, the correction failed. We then proceeded with the second technique of fully cutting the nail and then extracting it into two different pieces. The distal locking screw was removed, followed by removal of the proximal locking screw. Manual traction was applied as a distraction at the fracture site to fully visualise the apex of angulation of the bent nail. A Jumbo cutter was introduced up to the visible nail and it was sectioned by multiple bites into two parts as shown in Fig. 2a and b. Continuous saline irrigation was done during the transection of the nail. The proximal part of the nail was extracted with a standard technique using extraction nail system and the distal part was extracted through the fracture site using pliers. A bone graft was done and a locking compression plate was inserted to stabilize the fractured femur. (Fig 2c, d) Post-operative recuperation was uneventful and - the patient was discharged well without any complications.

## Discussion

A bent femoral intramedullary nail is an uncommon complication following secondary trauma to a fracture of the femur that is not united^3^. A bent titanium IM nail was identified to be the very difficult to manage. A broken nail can be easily removed through the fracture. However, a bent nail has to be broken before it can be removed. The first step in approaching the problem is a well-planned attempt to remove the deformed IM nail^4^. Few factors have to be identified before choosing the proper method to extract the nail which includes the implant material, nail thickness, degree of angulation on both projections, location of the apex, fracture pattern and last but not least, the quality of the bone (whether it is osteopenic or osteoporotic). The cost and availability of required tools or equipment, operating room conditions and the surgeon's expertise are also important. The next treatment options should be planned after removing the bent nail, which is either using an intra or extramedullary device^5^. If there is distal screw present, the distal screw should be removed first before cutting the nail.

Several studies and case reports outline the strategies and techniques for removing a bent IM nail. However, there is no gold standard protocol reported in the literature, and there is no single best method to remove a bent intramedullary nail. A few authors claimed that they have described novel techniques, but most shared similar steps with slight modifications^5^. There are in principle, five techniques of bent IM nail removal: (1) standard extraction with no additional intervention, (2) in situ straightening via external force on the femur followed by standard extraction, (3) partial weakening of the nail using high-speed burr and F tool followed by manual straightening, (4) fully sectioning of the nail using high-speed burr or metal cutter and extracting the nail in two pieces, and (5) using plate and reduction clamps to straighten the nail^5^. Most of these techniques and instruments are costly and not easily available in every setup. These procedures' expected complications include metal debris, thermal necrosis, and soft tissue damage, which can later affect the wound and fracture healing.

In this case, we used the technique based on the principle of its ability to fully cut the nail and extracting it in two pieces by using a Jumbo cutter^4^. This technique requires the skin incision around 2cm to 3cm at the fracture site to fully expose the bent nail. Then the distal locking screw was removed followed by removal of proximal locking screw. Manual traction was used to distract the fracture site in order to visualise the apex of angulation of the bent nail. A Jumbo cutter was used by making multiple bites at the visible nail and then it was sectioned into two parts. Continuous saline irrigation was done during the transection of the nail. The proximal part of the nail was extracted with standard technique using extraction nail system and the distal part was extracted through the fracture site using pliers. This technique is a simple, not technically demanding and cost-effective method without using expensive instruments. A Jumbo cutter is usually available in most operating rooms in most centres of the world. This technique also has a low level of complications, such as less soft tissue damage and without thermal necrosis. It is a rare and challenging orthopaedic problem to remove a bent intramedullary nail. Dealing with such cases requires technical tricks and a combination of strategies for the removal of a bent IM nail. The technique we used, in this case, was effective, based on the principles of its ability to fully cut the nail and extracting it in two pieces by using a Jumbo cutter. This technique is simple yet economical with the likelihood of causing less soft tissue damage and thermal necrosis.
